# Association of the Number of Teeth With Physical Function and Length of Hospital Stay After Hip Fracture Surgery: A Prospective Observational Study at a Tertiary Hospital in Japan

**DOI:** 10.7759/cureus.47297

**Published:** 2023-10-18

**Authors:** Kotaro Sorimachi, Nobuaki Moriyama, Satoshi Hatashita, Hisashi Miyajima, Shimpei Shigemoto, Kaori Takagi, Hiroko Hirano, Masayuki Ito, Ken Iseki, Seiji Yasumura

**Affiliations:** 1 Department of Public Health/Department of Emergency and Critical Care Medicine, Fukushima Medical University School of Medicine, Fukushima, JPN; 2 Department of Traumatology and Reconstructive Surgery, Aizu Chuo Hospital, Aizuwakamatsu, JPN; 3 Department of Public Health, Fukushima Medical University School of Medicine, Fukushima, JPN; 4 Department of Traumatology and Reconstructive Surgery, Fukushima Medical University, Fukushima, JPN; 5 Clinical Unit of Dentistry and Oral Surgery, Aizu Chuo Hospital, Aizuwakamatsu, JPN; 6 Rehabilitation Center, Aizu Chuo Hospital, Aizuwakamatsu, JPN; 7 Department of Nursing, Aizu Chuo Hospital, Aizuwakamatsu, JPN; 8 Department of Emergency and Critical Care Medicine, Fukushima Medical University School of Medicine, Fukushima, JPN

**Keywords:** hospital stay, muscle strength, physical function, number of teeth, hip fracture

## Abstract

Objectives: Although lower-extremity muscle strength is associated with physical function, there are challenges in assessing the muscle strength of patients after hip surgery due to pain or limited cognitive function. The number of teeth is a characteristic that can be easily examined. Although the relationship between the number of teeth and physical function has been reported in recent years, there are no reports examining the relationship with prognosis in patients with hip fractures. Therefore, this study aimed to investigate the relationship between the number of teeth and physical function and length of hospital stay after hip fracture surgery and to evaluate the predictive efficacy of the number of teeth on postoperative prognosis.

Methods: This prospective cohort study was conducted in a tertiary clinical care facility. Patients aged ≥65 years who underwent hip surgery were included. A total of 101 patients (mean age: 85.1±8.0 years) were included. The factor analyzed was the number of teeth at admission. Patients were divided into two groups according to the number of teeth: those with ≥20 and those with ≤19 teeth. The outcomes were knee extension muscle strength-to-weight ratio at two weeks postoperatively and the length of hospital stay. A multiple regression analysis was performed to determine the association between the two groups.

Results: Of 101 patients, 79 (78.2%) had ≤19 teeth, whereas 22 (21.8%) had ≥20 teeth. The mean muscle strength-to-weight ratio and length of hospital stay were 0.26±0.11 kgf/kg and 57.5±31.4 days, respectively. Multiple regression analysis revealed that the number of teeth was significantly associated with the muscle strength-to-weight ratio (β=-0.26, p=0.04) but not with the duration of hospitalization (β=0.17, p=0.09).

Conclusions: We suggest that assessment of the number of teeth at admission may be a useful predictor of patient physical function.

## Introduction

The population of Japan is aging progressively, and 30% of it is estimated to be ≥65 years of age by 2023, making Japan’s population the most aged worldwide [1]. Furthermore, the number of individuals requiring long-term care has been increasing, reaching 6.96 million in 2022, accounting for 19.1% of the population aged ≥65 years [2]. Dementia ranks as the most common cause of long-term care needs, whereas “fractures and falls” is the fourth most common cause [3].

Fractures in older adults, particularly lower-extremity fractures, require hospitalization and surgical treatment. Because the ambulatory function of older adult patients is considerably affected, they tend to be hospitalized for longer periods due to social factors and their bedridden state [4]. As Japan’s population ages, the number of older adults suffering from hip fractures is increasing, making it difficult to secure hospital beds, with patients needing inpatient care at risk of not receiving such care. Moreover, longer hospitalization periods lead to physical deterioration among older adult patients themselves. Accordingly, shortening the length of hospital stay (LOHS) of patients with hip fractures is appropriate.

Factors associated with postoperative physical function in patients with hip fractures include age, pre-existing disease, and cognitive function [5], whereas factors related to the LOHS include age, sex, lethargy, and smoking [6]. The presence of frailty (especially physical frailty) has been reported to prolong the LOHS [7]; nonetheless, this information is often uncertain because it relies on the memory of patients and their family members. Overall, using minimally invasive methods at the time of admission, reliable and conveniently obtained information that can define the degree of postoperative improvement in physical function is needed, however, is currently unavailable.

Recent reports have focused on the relationship between physical and oral function. Particularly, a reduction in the number of teeth has been applied as an indicator reflecting nutritional status, such as a decrease in body mass index (BMI) [8]. The number of teeth is also associated with physical frailty; the fewer the teeth, the greater the tendency toward frailty [9]. Counting the number of teeth is a simple and noninvasive method that may aid in predicting function in postoperative patients with hip fractures.

We aimed to investigate the relationship between the number of teeth, physical function, and postoperative LOHS among older adult patients hospitalized for hip fractures. Regarding the evaluation of physical function, we focused on gait function, which is also a Frail assessment item [9]. Since gait function has been reportedly related to lower limb muscle strength [10], we set lower limb muscle strength as a representative value of physical function. We also considered that a decrease in the number of teeth may be involved in prolonged hospitalization [7] via Frail status [9]. Therefore, we hypothesized that patients with fewer teeth would exhibit poorer postoperative lower limb muscle strength and have an extended postoperative LOHS than those with more teeth. We aimed to demonstrate the usefulness of counting the teeth in predicting the patients’ lower limb muscle strength and postoperative hospital stay after hip fracture surgery and subsequently to provide an in-hospital rehabilitation program matching the goal for each patient.

## Materials and methods

Study design and setting

This prospective cohort study (level of evidence: II) was conducted at Aizu Chuo Hospital (Fukushima, Japan), which serves as a tertiary clinical care center. The hospital has 713 beds, 11 operation rooms, and approximately 10,000 ward admissions per year. Approximately 200 hip fracture surgeries are performed by seven trauma and reconstructive surgeons, and the dedicated ward has 60 beds and 27 nurses. Additionally, this hospital possesses beds for acute and chronic care, thereby providing continuous acute to chronic care. Regarding hip fracture surgical techniques, osteosynthesis is selected for extra-articular fractures, while bipolar hip arthroplasty is used for intra-articular fractures. Typically, surgery is performed within 48 hours following the injury.

Participants

Patients aged ≥65 years who visited or were transported to General Hospital A after sustaining a hip fracture were included in this study. The exclusion criteria were patients who (i) had injuries caused by a traffic accident or other high-energy event; (ii) were bedridden or were using a wheelchair prior to the injury; (iii) were unable to undergo rehabilitation due to severe dementia; (iv) were unable to undergo early rehabilitation because of postoperative bed rest restriction; (v) were transferred to a hospital early after surgery; and (vi) died or in whom rehabilitative interventions could not be performed due to early postoperative transfer to another hospital or for other reasons.

Variables

The objective variables were lower-extremity muscle strength as a physical function and the LOHS. Lower-extremity muscle strength was assessed as knee-extension muscle strength, which has been reported to be related to walking ability [10] and was evaluated on postoperative week 2 [11]. Knee-extension muscle strength was measured using a strength analysis system (Isoforce GT-300© (margin of error: ±1% full scale); OG Wellness, Okayama, Japan) by the physical therapist assigned to the patient. Isometric knee extension muscle strength on the healthy side was recorded with the patients sitting on a chair with their knees flexed at 90º. Because postoperative pain may result in the inaccurate measurement of potential muscle strength in the affected lower extremity, muscle strength on the healthy side was recorded in this study. Measurements were obtained twice, each measuring the maximum muscle force within 10 seconds; the greater of the two measurements was taken as the patient’s knee-extension muscle force. Postoperative hospital stay was defined as the number of days from the date of surgery till discharge.

The main explanatory variable was the number of teeth. Herein, we investigated both the number of remaining teeth, irrespective of denture use, and the number of teeth when dentures were used. No distinction was made between natural and implant teeth when counting the remaining teeth. The data were obtained by a dental surgeon or hygienist.

The following covariates reported by previous studies to be associated with either physical function or the LOHS were adopted: age [5,12], sex, fracture type (femoral neck or trochanteric fracture), presence of other traumas at the time of injury, perioperative complications, surgical technique (osteosynthesis or bipolar hip arthroplasty [BHA]) [13], abnormal chest X-ray findings or [5,14] abnormal levels of hemoglobin [5,14] (cutoff value: 12 g/dL in men and 11 g/dL in women), albumin (cutoff value: 3.5 g/dL), and electrolytes [5], such as sodium (Na) (reference value: 135-146 mEq/L), potassium (K) (reference value: 3.4-5.1 mEq/L), and Cl (reference value: 99-110 mEq/L). Femoral neck and trochanteric fractures were classified into subcapital, transcervical, and basicervical subtypes and simple, multi-fragmentary, and intertrochanteric subtypes, respectively. Data on the abovementioned items were recorded by the patient’s physician and collected from the patient’s electronic medical records, and fracture type was classified by the physician in charge.

To respond to an aging society, the Japanese government implemented the national long-term care insurance (LTCI) system in 2000 to provide suitable care services through care-level assessment. Since then, LTCI services have been provided according to a certificate summarizing long-term care needs. There are seven levels of care-need certificates, starting with support levels 1 and 2 (mild disability), followed by care levels 1 and 2 (moderate disability), and care levels 3-5 (severe disability). According to a previous report [15], we categorized these levels into the following three categories: 1) none; 2) support levels 1 and 2; care levels 1 and 2; and 3) care levels 3-5. Cognitive function was assessed after admission using the revised version of Hasegawa’s Dementia Scale (HDS-R) [12,14]. These data were collected primarily by the ward nurses. To minimize bias, the outcome measurer was blinded to the number of teeth.

Sample size

The number of study participants was estimated based on a previous report [16], with eight explanatory variables for the multiple regression analysis. Given that at least 10 individuals are required for each explanatory variable according to a previous study [17], data for 80 participants were required. Considering difficulties in measurement, a 50% dropout rate at follow-up was estimated, and the number of study participants was set at 160. The survey was terminated when the number of study participants reached the target sample size.

Statistical analysis

The muscle strength-to-weight ratio was calculated by dividing the measured value by body weight to eliminate the effect of body weight on the objective variable (i.e., knee extensor strength) [18]. To identify the effect of the number of teeth at admission on physical function and the postoperative LOHS, we compared the muscle strength-to-weight ratio on the healthy side and the postoperative LOHS in each group, with the number of teeth categorized into “≥20” and “≤19” based on methodologies previous studies [19].

The association between explanatory and outcome variables was examined using Pearson’s correlation coefficient or t-test. Nonparametric tests were performed when the variables were not normally distributed. A multiple regression analysis was performed to investigate the association between the number of teeth and the muscle strength-to-weight ratio on the healthy side at two postoperative weeks and the length of postoperative LOHS.

The covariates age and sex were used as basic data in the analysis of the number of teeth. Previous studies identified cognitive function as a predictor of walking ability at the time of discharge from the hospital [14] and demonstrated an association between the surgical technique for hip fractures and postoperative walking ability [13]. Hence, the HDS-R score and surgical technique were also used as covariates, with an HDS-R score of ≤20 points indicating the presence of dementia as a categorical variable. Accordingly, the covariates to be adjusted for were age, sex, surgical technique (osteosynthesis or BHA), and HDS-R score (≥21 points or <21 points).

As for the covariates considered in the analysis of postoperative hospital stay, age, and sex were utilized as basic data; the surgical technique and place of residence (home or facility) were also used based on previous studies.

All statistical analyses were performed by the corresponding author using Stata SE version 17 (StataCorp LLC, College Station, Texas, USA) with a significance level of 5%. No special completion was performed for missing data. For each item, the analysis was performed on participants with data for that item.

Ethical consideration

This study adhered to the principles of the Declaration of Helsinki and was approved by the Institutional Review Board of Fukushima Medical University (approval no.: General 2021-211; 9 November 2021). Patients and their families were informed of the study upon admission and were requested to sign an informed consent form before their study participation.

## Results

Participants

Patients transported to our hospital with a hip fracture from November 29, 2021, to November 18, 2022, were analyzed in this study. In total, 145 patients met the inclusion criteria and provided informed consent for the study.

**Figure 1:**
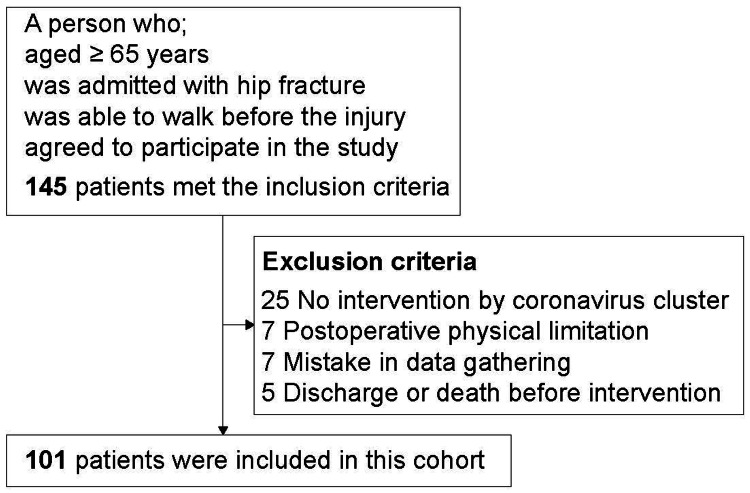
Participant flow

Among these 145 patients, 25 were dropped because they could not be rehabilitated due to an outbreak of ward clusters caused by a novel coronavirus infection; all these patients were admitted prior to the outbreak. Seven, seven, and five patients were dropped due to bed rest restriction, because their information was not obtained, and due to early transfer or early death prior to study commencement, respectively. Accordingly, the final study population comprised 101 patients (Figure 1).

Descriptive data

Of the 101 study participants, 31 (30.7%) were male, and 70 (69.3%) were female. The mean patient age was 85.1±8.0 years, with 14 (13.9%) and 87 (86.1%) patients being ≤74 years and ≥75 years of age, respectively. Furthermore, 42 (41.6%), 57 (56.4%), and two (2.0%) patients sustained femoral neck, trochanteric, and subtrochanteric fractures, respectively. The surgical techniques were BHA and osteosynthesis in 44 (43.6%) and 57 (56.4%) patients, respectively.

Regarding the number of teeth, 79 (78.2%) participants had ≤19 teeth, and 22 (21.8%) participants had ≥20 teeth; 64 (63.4%) patients were denture users. When the participants were divided into two groups according to the number of teeth at the time of denture use, 79 (78.2%) and 22 (21.8%) patients were in the ≥20 and ≤19 teeth groups, respectively (Table 1).

**Table 1:** Basic data on study participants according to sex The following were examined: existing diseases (diabetes, heart failure, arrhythmia, ischemic heart disease, cerebrovascular disease, renal disease, tumor, Parkinson’s disease, treatment with antiplatelet agents, hypertension, or chronic obstructive pulmonary disease); corticosteroid use; type of residence (home or facility); family living status (single, with a spouse only, or with others); marital status (never married, married, or bereaved); drinking history (nondrinker, occasional drinker, or daily drinker); smoking habits (no, used to smoke, or yes); walking ability prior to injury (independent (with or without the use of aids), assisted (with or without the use of aids), wheelchair, or bedridden); and exercise habits prior to injury (exercise for ≥30 minutes at least twice a week for at least one year). †: The Revised Hasegawa's dementia scale

			Male (n=31)	Female (n=70)	Overall (n=101)
Age (years)		Mean (SD)	84.2(8.6)	85.5(7.8)	85.1(8.0)
	≧75	n(%)	26(83.9)	61(87.1)	87(86.1)
	<75		5(16.1)	9(12.9)	14(13.9)
BMI (kg/m^2^)	<18.5	n(%)	5(16.1)	18(25.7)	23(22.8)
	≤18.5, <25		17(54.8)	40(57.1)	57(56.4)
	≤25		9(29.1)	12(17.2)	21(20.8)
Existing disease	Yes	n(%)	26(83.9)	60(85.7)	86(85.2)
Corticosteroid use	Yes	n(%)	0(0)	2(2.9)	2(2.0)
Level of care	None	n(%)	12(38.7)	19(27.1)	31(30.7)
	Requiring help - long-term care level 2		12(38.7)	37(52.9)	49(48.5)
	Long-term care levels 3-5		7(22.6)	14(20)	21(20.8)
Residence	Home	n(%)	23(74.2)	58(82.9)	81(80.2)
	Facility		8(25.8)	12(17.1)	20(19.8)
Family living status	Single	n(%)	3(9.7)	9(12.9)	12(11.9)
	With a spouse only		9(29.0)	7(10)	16(15.8)
	Others		19(61.3)	54(77.1)	73(72.3)
Marital status	Never married	n(%)	2(6.4)	1(1.4)	3(3.0)
	Married		22(71.0)	31(44.3)	53(52.5)
	Bereaved		7(22.6)	38(54.3)	45(44.5)
Drinking history	Nondrinker	n(%)	23(74.2)	68(97.2)	91(90.1)
	Occasional drinker		3(9.7)	1(1.4)	4(4.0)
	Daily drinker		5(16.1)	1(1.4)	6(5.9)
Smoking habits	No	n(%)	25(80.7)	68(97.2)	93(92.1)
	Used to smoke		5(16.1)	1(1.4)	6(5.9)
	Yes		1(3.2)	1(1.4)	2(2.0)
Walking ability prior to injury	Independent without aids	n(%)	18(58.1)	34(48.6)	52(51.5)
	Independent with aids		9(29.0)	21(30)	30(29.7)
	Assisted without aids		1(3.2)	6(8.6)	7(6.9)
	Assisted with aids		3(9.7)	9(12.8)	12(11.9)
Exercise habits prior to injury	Yes	n(%)	3(9.7)	9(12.9)	12(11.9)
HDS-R score^†^	≥21	n(%)	11(35.5)	31(44.3)	42(41.6)
	≤20		20(64.5)	39(55.7)	59(58.4)
Abnormal findings on X-ray	Yes	n(%)	4(12.9)	9(12.9)	13(12.9)
Hemoglobin level	Low	n(%)	15(48.4)	20(28.6)	35(34.7)
Electrolyte level	Abnormal	n(%)	8(25.8)	10(14.3)	18(17.8)
Albumin level	Low	n(%)	12(38.7)	17(24.3)	29(28.7)
Fracture type	Femoral neck fracture	n(%)	14(45.2)	28(40.0)	42(41.6)
	Subcapital		6(42.9)	9(32.1)	15(35.7)
	Transcervical		8(57.1)	18(64.3)	26(61.9)
	Basicervical		0(0)	1(3.6)	1(2.4)
	Trochanteric fracture		17(54.8)	42(60.0)	59(58.4)
	Simple pertrochanteric		10(58.8)	8(19.0)	18(30.5)
	Multifragmentary pertrochanteric		6(35.3)	33(78.6)	39(66.1)
	Intertrochanteric		1(5.9)	1(2.4)	2(3.4)
Surgical technique	Bipolar hip arthroplasty	n(%)	16(51.6)	28(40.0)	44(43.6)
	Osteosynthesis		15(48.4)	42(60.0)	57(56.4)
Other traumas	Yes	n(%)	1(3.2)	2(2.9)	3(3.0)
Perioperative complications	Yes	n(%)	10(32.3)	23(32.9)	33(32.7)
Number of teeth		Median (IQR)	5(1-21)	4.5(0-15)	5(0-16)
	≥20	n(%)	8(25.8)	14(20.0)	22(21.8)
	≤19		23(74.2)	56(80.0)	79(78.2)
Denture use	Yes	n(%)	15(48.4)	49(70.0)	64(63.4)
Number of teeth after denture use		Median (IQR)	25(13-28)	28(23-28)	27(22-28)

Univariate analysis of basic data between the two groups for the number of teeth showed significant differences in age (p=0.004, 95% confidence interval (CI) (-9.14, -1.74)), anemia (p<0.001), fracture type (p=0.018), surgical technique (p=0.008), and denture use (p<0.001) (Table 2).

**Table 2:** Basic data on study participants according to the number of teeth †: t-test ‡: The Revised Hasegawa’s dementia scale

			Number of teeth		p-value
			≧20	20＞	
Age		Mean (SD)	80.9(6.8)	86.3(8.0)	^†^0.004
	≥75	n(%)	19(86.4)	68(86.1)	
	<75		3(13.6)	11(13.9)	0.972
Sex	Male	n(%)	8(36.4)	23(29.1)	0.514
BMI	<18.5		5(22.7)	18(22.8)	
	≥18.5, <25		10(45.5)	47(59.5)	
	≥25	n(%)	7(31.8)	14(17.7)	0.325
Existing disease	Yes	n(%)	19(86.4)	67(84.8)	0.856
Corticosteroid use	Yes	n(%)	1(4.6)	1(1.3)	0.329
Level of care	None		10(45.4)	21(26.6)	
	Requiring help - long-term care level 2		8(36.4)	41(51.9)	
	Long-term care levels 3-5	n(%)	4(18.2)	17(21.5)	0.229
Residence	Home		18(81.8)	63(79.8)	
	Facility	n(%)	4(18.2)	16(20.3)	0.829
Family living status	Single		3(13.6)	9(11.4)	
	With a spouse only		5(22.7)	11(13.9)	
	Others	n(%)	14(63.6)	59(74.7)	0.547
Marital status	Never married		1(4.6)	2(2.5)	
	Married		15(68.2)	38(48.1)	
	Bereaved	n(%)	6(27.3)	39(49.4)	0.179
Drinking history	Nondrinker		18(81.8)	73(92.4)	
	Occasional drinker		1(4.6)	3(3.8)	
	Daily drinker	n(%)	3(13.6)	3(3.8)	0.218
Smoking habits	None		21(95.5)	72(91.1)	
	Used to smoke		0(0)	6(7.6)	
	Yes	n(%)	1(4.6)	1(1.3)	0.267
Walking ability prior to injury	Independent without aids		13(59.1)	39(49.4)	
	Independent with aids		3(13.6)	27(34.2)	
	Assisted without aids		2(9.1)	5(6.3)	
	Assisted with aids	n(%)	4(18.2)	8(10.1)	0.274
Exercise habits prior to injury	yes	n(%)	5(22.7)	7(8.9)	0.075
HDS-R score^‡^	≥21		10(45.5)	32(40.5)	
	≤20	n(%)	12(54.5)	47(59.5)	0.677
Abnormal findings on X-ray	Yes	n(%)	2(9.1)	11(13.9)	0.549
Hemoglobin level	Low	n(%)	0(0)	35(44.3)	<0.001
Electrolyte level	Abnormal	n(%)	4(18.2)	14(17.7)	0.960
Albumin level	Low	n(%)	4(18.2)	25(31.7)	0.217
Fracture type	Femoral neck fracture		14(63.6)	28(35.4)	
	Trochanteric fracture	n(%)	8(36.4)	51(64.6)	0.018
Surgical technique	Bipolar hip arthroplasty		15(68.2)	29(36.7)	
	Osteosynthesis	n(%)	7(31.8)	50(63.3)	0.008
Other traumas	Yes	n(%)	1(4.6)	2(2.5)	0.623
Perioperative complications	Yes	n(%)	7(31.8)	26(32.9)	0.924
Denture use	Yes	n(%)	3(13.6)	61(77.2)	<0.001

Outcome data

Knee extension muscle strength on the healthy side was measured in 65 study participants, excluding those who could not be weaned off the bed even at two weeks postoperatively and whose muscle strength could not be measured. The postoperative LOHS was calculated for 98 study participants, excluding three participants who were still hospitalized at the end of this study. The mean muscle strength-to-weight ratio and mean postoperative LOHS were 0.26±0.11 kgf/kg and 57.5±31.4 days, respectively. The intraclass correlation coefficient (ICC (1,1)) for the muscle-strength measurement was 0.95, which has sufficiently high reliability.

Table 3 presents the results of the comparison between the two groups regarding the number of teeth. The results of the t-test indicated that only the muscle strength-to-weight ratio was significantly higher in the group with ≥20 teeth than in the group with ≤19 teeth (p=0.011, 95% CI (0.02, 0.15)). No significant difference in the length of hospitalization was observed (p=0.058, 95% CI (-29.8, 0.50)).

**Table 3:** Comparison of outcome data according to the number of teeth

			Number of teeth		
			≥20	<19	p-value
Muscle strength-to-weight ratio	[kgf/kg]	n	12	53	
		Median (IQR)	0.28(0.27-0.42)	0.24(0.20-0.29)	0.011
Postoperative hospital stay	[day]	n	21	77	
		Median (IQR)	37(28-66)	55(38-76)	0.058

Multiple regression analysis of the muscle strength-to-weight ratio

Univariate analysis revealed that the following items were related: age, level of care, drinking history, walking ability before the injury, HDS-R score, number of teeth, and denture use. Caregiver certification, denture use, drinking history, and walking ability before injury were excluded from the adjustment variables because of a significant bias in the number of cases.

The results of multiple regression analysis indicated that the muscle strength-to-weight ratio was statistically significantly higher in participants with more teeth (β=-0.26, 95% CI (-0.14, 0.00), p=0.04). In the subgroup analysis, sex was excluded from the aforementioned model as a covariate. However, no statistically significant association was found (male: β=-0.19, 95% CI (-0.17, 0.07), p=0.39; female: β=-0.26, 95% CI (-0.16, 0.02), p=0.10) (Table 4).

**Table 4:** Multiple regression analysis of the muscle strength-to-weight ratio (overall and according to sex) †: Partial regression coefficient ‡: Standardized partial regression coefficient §: The Revised Hasegawa's dementia scale ¶; Bipolar hip arthroplasty

		Overall (n=65)				Male (n=22)				Female (n=43)			
	Reference	B^†^	Β^‡^	p-value	95% CI	B	β	p-value	95% CI	B	β	p-value	95% CI
Number of teeth	≥20	-0.07	-0.26	0.04	-0.14, 0.00	-0.05	-0.19	0.39	-0.17, 0.07	-0.07	-0.26	0.10	-0.16, 0.02
Age	<75	0.01	0.05	0.72	-0.06, 0.09	-0.06	-0.16	0.52	-0.24, 0.12	0.03	0.12	0.46	-0.06, 0.13
Sex	Male	-0.01	-0.05	0.71	-0.06, 0.04								
Surgical technique	BHA^¶^	0.01	0.06	0.64	-0.04, 0.07	0.04	0.21	0.36	-0.05, 0.14	-0.01	-0.04	0.84	-0.09, 0.07
HDS-R score^§^	≥21	-0.08	-0.38	<0.01	-0.14, -0.03	-0.08	-0.37	0.12	-0.18, 0.02	-0.07	-0.32	0.05	-0.14, 0.00
		Adj R-squared=0.15				Adj R-squared=0.09				Adj R-squared=0.14			

Multiple regression analysis of the postoperative hospital stay

Univariate analysis was performed to identify basic data items associated with a postoperative LOHS, and the following items were found to show an association: the level of care, marital and drinking history, fracture type, surgical technique, and the number of teeth. Marital and drinking history were excluded from the covariates because of the bias in the number of cases. Level of care and HDS-R score, which were associated with residence in the univariate analysis, were excluded from the variables.

The results of multiple regression analysis indicated no statistically significant association with postoperative hospital stay (β=0.17, 95% CI (-1.92, 28.0), p=0.09). Similarly, no association was identified in the subgroup analysis by sex (male: β=0.06, 95% CI (-21.7, 30.4), p=0.74; female: β=0.20, 95% CI (-3.46, 34.4), p=0.11) (Table 5).

**Table 5:** Multiple regression analysis of the length of postoperative hospital stay (overall and according to sex) †: Partial regression coefficient  ‡: Standardized partial regression coefficient §: Bipolar hip arthroplasty

		Overall (n=98)				Male (n=29)				Female (n=69)			
	Reference	B^†^	Β^‡^	p-value	95% CI	B	β	p-value	95% CI	B	β	p-value	95% CI
Number of teeth	≥20	13.03	0.17	0.09	-1.92, 28.0	4.31	0.06	0.74	-21.7, 30.4	15.48	0.20	0.11	-3.46, 34.4
Age	<75	25.40	0.28	0.01	7.51, 43.3	41.01	0.53	0.01	11.8, 70.2	17.53	0.19	0.15	-6.27, 41.3
Sex	Male	4.60	0.07	0.49	-8.47, 17.7								
Surgical technique	BHA^§^	4.97	0.08	0.46	-8.28, 18,2	-5.89	-0.10	0.60	-28.8, 17.1	11.60	0.18	0.17	-5.18, 28.4
Residence	Home	-15.78	-0.20	0.04	-30.5, -1.02	-22.06	-0.34	0.08	-46.7, 2.54	-13.82	-0.17	0.16	-33.0, 5.38
		Adj. R-squared=0.14				Adj. R-squared=0.19				Adj. R-squared=0.11			

## Discussion

Herein, we demonstrated an association between the number of teeth in patients with hip fractures and the lower-extremity muscle strength-to-weight ratio at two weeks postoperatively (p=0.04), suggesting that ascertaining the number of teeth at the time of delivery may predict muscle strength and, consequently, the physical function level at two weeks postoperatively. This result supported our initial hypothesis. A previous study reported an association between the number of teeth and muscle strength, as well as a significant association between grip strength and the number of teeth in healthy participants [20]. Our study supports the results of that study by showing the association between the number of teeth and muscle strength in patients with hip fractures.

Several mechanisms may explain the association between the number of teeth and muscle strength. First, nutritional status may be related. Srisilapanan et al. reported a significant association between having fewer teeth and a lower BMI in older adults and identified BMI as an indicator of overall nutritional status [8]. Shin HS demonstrated that the more teeth a person has, the higher their diet quality [21], thereby confirming that nutritional intake changes with the number of teeth. Furthermore, Saarela et al. demonstrated that the worse a person’s dental condition is, the more malnourished they are [22]. Furuta et al. reported that a decrease in the number of teeth led to a decrease in swallowing function and the suppression of saliva secretion [23]. In this study, albumin levels were measured as an indicator of nutritional status, and the univariate analysis showed an association between the number of teeth and albumin levels (p=0.012). These results suggest that, as a dental condition, the number of teeth may be explained by a mechanism influencing nutritional status, which is related to muscle strength. Clearly, a healthy nutritional status is crucial for maintaining muscle mass and strength [24].

Second, the number of teeth is related to the bite. Grosdent et al. confirmed the relationship between the bite strength of the teeth and knee extension strength and reported that the muscle strength was reduced by an imbalance in the bite [25]. Hence, the group with fewer teeth in this study may have had a greater occlusal imbalance, resulting in lower muscle strength. Various reports have focused on dental occlusion; particularly, dental malocclusion causes dysfunctions in immunity and central catecholaminergic neurotransmission [26] and is implicated in the development of delirium [27]. While it remains unclear how the bite is directly involved in the maintenance of muscle strength, these reports clearly indicate a relationship between tooth occlusion and muscle strength, and muscle strength is possibly reduced as a result of inadequate occlusion attributable to a small number of teeth.

This study demonstrated that examining the number of teeth in patients with hip fractures could predict postoperative lower-extremity muscle strength clinically. Previous studies reported the association of lower-extremity muscle weakness with the risk of hospitalization and death [28]. Other factors, such as increased sitting time during hospitalization and increased frailty, have also been reported to indicate physical weakness [29]. Accordingly, the presence of lower-extremity muscle weakness is a predictor of walking ability, and the presence of muscle weakness is closely related to a reduction in walking ability. Even if muscle strength cannot be measured due to circumstances, observing the oral condition may provide some surrogate assessment. This result may also suggest a good practice of interprofessional collaboration to predict postoperative physical function in patients with hip fractures.

The present study also demonstrated the absence of an association between the number of teeth and postoperative hospital stay duration, which did not support our initial hypothesis. Age, female sex, absence of a dating partner, being a smoker, degree of complications, admission from another hospital, weekend hospitalization, and several hospitalizations in the past 12 months have been recognized as factors contributing to long-term hospitalization [6]. Recently, surgical intervention for hip fractures has been recommended as an early surgical intervention and has been reported to prolong hospital stay and increase mortality in patients treated 48 hours after the injury [30]. In our hospital, we followed this recommendation and performed surgical intervention within two days of a patient’s arrival; therefore, there was no difference in the timing of surgical intervention.

One reason for the lack of statistically significant results is that several socially hospitalized patients were physically able to be discharged from the hospital but could not be discharged early because of the hospital’s acceptance arrangements and other reasons [4]. The hospital where this study was conducted has not only acute care beds but also chronic care beds; hence, patients are transferred within the hospital rather than being transferred to other hospitals, and chronic care management is continued until discharge. Therefore, the postoperative LOHS was prolonged, and it is likely that social factors, such as the inability to find a place to be discharged, had an impact on the l postoperative LOHS. Murphy et al. reported that early surgical intervention within 48 hours resulted in a significantly shorter hospital stay [30]. Regarding hospital stay, the present study reported 12.6±6.1 and 15.5±10.1 days for the ≤24 and ≥48 hours groups, respectively. Given that the postoperative LOHS was 57.5±31.4 days in the present study as well, the LOHS tends to be clearly longer in Japan than in other countries. Accordingly, the contribution of the number of teeth may have been reduced as a result of the involvement of non-adjustable factors aside from physical function in the postoperative LOHS.

This is the first study to focus on patients with hip fractures, which are a frequent occurrence, and to investigate the association between the number of teeth and postoperative outcomes in these patients, thereby revealing that the number of teeth may possibly predict postoperative physical function.

Nonetheless, this study had some limitations. First, as this study was conducted at a single institution in Japan, there might have been biases in the functional characteristics of the hospital and the content of rehabilitative interventions, which may not be generally applicable. Second, data on patients who could not be weaned off the bed during rehabilitation due to pain or other reasons and whose muscle strength could not be measured were missing; thus, the muscle strength values might have been overestimated. Finally, only a few male study participants were included due to the nature of the disease.

## Conclusions

We found that the number of teeth in patients after hip fracture surgery was associated with lower-extremity muscle strength at two weeks postoperatively and the number of teeth showed no association with the postoperative LOHS. Assessing the number of teeth at admission may be helpful in predicting a patient’s physical function. The results can be used to select a discharge site and plan necessary rehabilitation at an early stage, thereby reducing the hospital stay.
